# 17β-estradiol maintains extracellular matrix homeostasis of nucleus pulposus cells by activating p70 S6K1 signaling pathway

**DOI:** 10.3389/fcell.2025.1564458

**Published:** 2025-06-06

**Authors:** Tao Liu, Zhaohui Li, Wei Zhang, Xuzhao Guo, Guobin Liu, Dalong Yang, Sidong Yang

**Affiliations:** ^1^ Department of Spinal Surgery, Hebei Medical University Third Hospital, Shijiazhuang, China; ^2^ Department of Orthopaedic Surgery, Hebei General Hospital, Shijiazhuang, China; ^3^ Department of Orthopaedic Surgery, Hebei Children’s Hospital, Shijiazhuang, China; ^4^ Department of Orthopedic Surgery, First Hospital of Hebei Medical University, Shijiazhuang, China; ^5^ Department of Orthopedic Surgery, Hebei Medical University Third Hospital, Shijiazhuang, China

**Keywords:** intervertebral disc degeneration, nucleus pulposus, estradiol, P70 S6K1, mTOR, apoptosis

## Abstract

**Background:**

Estrogen can inhibit the apoptosis of nucleus pulposus cells (NPCs) through the PI3K/AKT/mTOR signaling pathway. However, the downstream of mTOR signaling pathway remains elusive. This study investigates the effect of 17β-estradiol (E2) on intervertebral disc degeneration (IVDD) through the p70 S6K1 signaling pathway, downstream of mTOR.

**Methods:**

The IVDD model of rats was established by needle puncture and bilateral ovariectomy. Fifteen Sprague-Dawley rats were randomly assigned to the following three groups: (A) Sham surgery group (Sham); (B) Bilateral ovariectomy, 21G needle puncture and carrier injection (OVX + veh); (C) Bilateral ovariectomy, 21G needle puncture, E2 supplementation (OVX + E2). The degree of IVDD was evaluated by X-ray, magnetic resonance imaging (MRI), hematoxylin and eosin (H&E), and Safranin O-Fast Green staining. The expression levels of target protein p70S6K1 and its phosphorylated products were detected by immunohistochemistry (IHC). Finally, Western blot analysis and immunofluorescence staining were used to investigate the effect of E2 on the p70 S6K1 signaling pathway *in vitro*.

**Results:**

Histological staining and radiological results showed that E2 supplementation altered signaling, suggesting that it may have a protective effect against IVDD. IHC showed that compared with the Sham and OVX + E2 groups, the level of p70 S6K1 in the OVX + veh group was significantly increased while the expression of phosphorylated products (p-S6) was significantly decreased, suggesting that E2 could inhibit IVDD by activating p70 S6K1 signaling pathway, the downstream of mTOR. Furthermore, cellular immunofluorescence and Western blot showed that E2 can maintain extracellular matrix (ECM) balance and inhibits apoptosis of nucleus pulposus cells (NPCs) by activating the p70 S6K1 signaling pathway.

**Conclusion:**

In summary, 17β-estradiol mitigates IVDD progression by maintaining ECM homeostasis and inhibiting NPCs apoptosis through activation of the p70 S6K1 signaling pathway downstream of mTOR.

## 1 Introduction

In recent years, many studies have reported that intervertebral disc degeneration (IVDD) is one of the leading causes of chronic low back pain (Vergroesen et al., 2015; Vlaeyen et al., 2018; Chou et al., 2011; Luoma et al., 2000). Due to the disability of low back pain, the labor force decreases, increasing the medical cost (Andersson, 1999; Katz, 2006). These have brought a heavy economic burden to human society (Prince et al., 2015). The pathogenesis of IVDD may be related to factors such as age, genetic susceptibility, spinal instability, inflammation, increased ECM degradation, and abnormal NPC apoptosis (Parreira et al., 2018).

The intervertebral disc (IVD) has a complex structure. It is mainly composed of three parts: the nucleus pulposus (NP), the annulus fibrosus (AF), and the cartilaginous endplates (Urban and Roberts, 2003). The NP is the central part of the IVD and consists of nucleus pulposus cells (NPCs) and a large amount of extracellular matrix (ECM) (Adams et al., 2009; Sive et al., 2002; Zhang et al., 2016). The ECM mainly comprises NPCs, which secrete aggrecan and Collagen II, thus maintaining the typical structure of the IVD. NPCs are susceptible to oxidative stress, cellular senescence, inflammation, and apoptosis. When NPCs are reduced, this can lead to IVDD. As IVDD progresses, inflammatory factors such as IL-1β are increased in NP tissues. While IL-1β increases, disintegrin-like ADAM metallopeptidases (ADAMTS) with thrombochondrotin type 1 motifs and matrix metalloproteinases (MMPs) damaging ECM also increase (Wang et al., 2019). Previous studies have found that the MMPs family is a critical factor in the catabolism of ECM and plays a crucial role in the pathological process of IVDD (Molinos et al., 2015; Zawilla et al., 2014; Zhang et al., 2013). Recent studies have shown that apoptosis and inflammation of NPCs are the leading causes of the pathological process of IVDD (Ding et al., 2013; Risbud and Shapiro, 2014).

Some studies have found that estrogen regulates the development and progression of IVDD by regulating cell activity and ECM metabolism (Wang et al., 2016; Jin et al., 2018). At the same time, clinical studies have found that the incidence of IVDD in older women is higher than that in older men, which may be closely related to the decrease of estrogen levels in postmenopausal women (Wang, 2015; Wang, 2017). Estrogen plays a vital role in inhibiting inflammatory factors, oxidative stress, and excessive aging by binding to estrogen receptors (ERs) (Song et al., 2014; Santos et al., 2017). Previous studies have shown that 17β-estradiol (E2) has a significant inhibitory effect on the apoptosis of NPCs, and E2 can alleviate the progression of IVDD by downregulating the expression of MMPs (Santos et al., 2017; Kim et al., 2014; Zhou et al., 2015; Rosner et al., 2012). However, the mechanism by which E2 inhibits NPCs apoptosis and inflammation to improve IVDD remains unclear.

mTOR forms signaling pathways with downstream essential signaling proteins S6K1 and 4E-BP1, respectively, and regulates the apoptotic process. Kim et al. (2014) reported that Brassinin can induce human PC-3 prostate cancer cell apoptosis by inhibiting the PI3K/Akt/mTOR/S6K1 signaling pathway. Zhou et al. (2015) found that rotenone can induce hydrogen peroxide generation and inhibit mTOR-mediated S6K1 and 4E-BP1/eIF4E signaling pathways, thus leading to neuronal cell apoptosis. It is known that NPCs apoptosis is regulated by various pathways, among which inhibition of the PI3K/AKT/mTOR signaling pathway is one of the mechanisms leading to human IVDD (Rosner et al., 2012; Kakiuchi et al., 2019; Chen et al., 2020). Previous studies have confirmed that E2 can inhibit NPCs apoptosis through the PI3K/AKT/mTOR signaling pathway (Bai et al., 2021). However, the effect of the downstream mTOR signaling pathway on the IVD is unclear. Therefore, this study investigated whether E2 inhibited IL-1β-induced NPCs apoptosis through the p70S6K1 signaling pathway downstream of mTOR.

## 2 Methods

### 2.1 Ethics statement

The rats used in this study were purchased from the Animal Experimental Center of Hebei Medical University, and the animal work was approved by the Institutional Animal Care and Use Committee of the Hospital (No. Z2022-023-1).

### 2.2 IVDD models

According to our previous studies, needle puncture was used to establish rat coccygeal IVDD models (Liu et al., 2018; Wang et al., 2021). Fifteen Sprague-Dawley rats (280–330 g, 3 months old, female) were randomly assigned to the following three groups. (A) Sham group (Sham); (B) Bilateral ovariectomy, 21G needle puncture and carrier injection (OVX + veh); (C) Bilateral ovariectomy, 21G needle puncture, E2 supplementation (OVX + E2). After anesthesia, all rats were placed in the prone position. The Co7/8 and Co8/9 discs of the rat tails were located by finger palpation. After disinfecting the tail skin area, a 22G blade was used to incise the skin into the deep fascia. A 21G sterile needle was inserted into the IVD for 4.0mm, rotated 180°, left for 15 s, and removed. Finally, the incision was sutured. The discs of Co7/8 and Co8/9 were punctured in the OVX + veh and OVX + E2 groups, while only the skin of Co7/8 and Co8/9 was incised and sutured in the Sham group. At the same time, the OVX + veh and OVX + E2 groups had both ovaries removed, while the Sham group only had a small amount of fat removed.

The OVX + E2 group received a subcutaneous injection of E2 (10 μg/kg/day, E8875, Sigma-Aldrich, MO, USA) dissolved in corn oil (HYY 1888, MCE) (Wang et al., 2021). In contrast, the Sham and OVX + veh groups only received a subcutaneous injection of corn oil. All rats were placed in a ventilated environment at a constant temperature of 21°C with a light-dark cycle time of 12:12 h. After 4 weeks, X-ray and MRI scans were performed on all rats. Rats were then euthanized by an overdose of sodium pentobarbital (100 mg/kg, intraperitoneal injection; Sigma-Aldrich, P3761) followed by cervical dislocation to ensure death. Finally, tail IVD samples were separated and fixed in 4% paraformaldehyde (PFA) (Beyotime Biotechnology, China, P0099) for 48 h, followed by decalcification with 10% EDTA (PH = 7.25) for 30 days. All IVDs were embedded in paraffin and midsagittally sectioned to slices of 5 μm thickness.

### 2.3 The radiograph and MRI analysis

All rats were examined by X-ray before and 4 weeks after the surgery. The radiological images were obtained from the X-ray system (Epson Perfection V750 Pro; Long Beach, CA) and measured using MicroDicom viewer software (version 2.9.0). Disc height index (DHI) was used to assess disc height loss (Han et al., 2008). MRI images were performed on the system (Agilent 7 T/R16, CA). The degree of IVDD was evaluated using the modified Thompson scale (Masuda et al., 2005), which was based on signal intensity ranging from grade 1 to grade 4 (1 = normal, 2 = minimal decrease in signal intensity but the apparent narrowing of high-signal area, 3 = moderate decrease in signal intensity, and 4 = severe decrease in signal intensity).

### 2.4 Hematoxylin and eosin (H&E) and Safranin O-Fast Green staining

The rat tail vertebrae (including IVD) were separated from the adjacent vertebrae, fixed with 4% PFA for 48 h, washed with PBS, and decalcified with 10% EDTA (PH = 7.25) for 30 days. All IVDs were embedded in paraffin wax and cut into 5 μm midsagittal sections.

Sections were procedurally dewaxed and dehydrated, then stained with H&E (Solarbio, Cat^#^G1120, China) and Safranin O-Fast Green (Solarbio, Cat^#^G1371, China), respectively. Digital images were obtained using an optical microscope (BX53F2, Olympus, Japan). The histological grading of disc degeneration is as follows: five features of disc structure (cell structure of annulus fibrosus, morphology of annulus fibrosus, boundary between annulus fibrosus and nucleus pulposus, cellularity of nucleus pulposus and morphology of nucleus pulposus) were analyzed, and the degree of degeneration was evaluated on a 3-point scale, with five indicating normal IVD and 15 indicating severe disc degeneration (Han et al., 2008).

### 2.5 Immunohistochemistry

The preparation of tissue sections was conducted as above. Next, the slices were dewaxed and rehydrated, and then the antigen was repaired with 0.1% EDTA-free pancreatic enzyme repair solution in a 37°C oven for 1 h. The rabbit two-step assay kit (ZSBG-BIO, Beijing, China) was used for immunohistochemistry (IHC). The appropriate amount of endogenous peroxidase blocker was used to block endogenous peroxidase for 10 min. Then, the sections were incubated with the following primary antibodies at 4°C overnight: p70 S6K1 (1:200, 14485-1-AP, Proteintech), Phospho-S6 (Ser240/244) (1:1000, D68F8, Cell Signaling Technology), MMP3 (1:100, 17873-1-Ap, Proteintech), Aggrecan (1:100, DF7561, Affinity), Collagen II (1:100, AF0135, Affinity), Cleaved caspase-3 (1:400, 9661s, Cell Signaling Technology). After rewarming for 1h, tissue slices were added with an enhancing solution and incubated at 37°C for 20 min after cleaning. After cleaning, the tissue sections were added with enhanced enzyme-labeled goat anti-rabbit IgG polymer and incubated at 37°C for 20 min. DAB color developed at room temperature for 5–8 min. Finally, cell nuclei were stained with hematoxylin, dehydrated with gradient alcohol, and sealed with xylene transparent and neutral gum. All images were taken under an optical microscope. ImageJ software (version 1.53k; National Institutes of Health, Bethesda, MD, USA) was used to measure the average optical density (IOD value/Area) for semi-quantitative analysis.

### 2.6 Cell culture

We purchased human nucleus pulposus cells (Cat NO: CP-H097) from Procell Life Science& Technology (Wuhan, China) and cultured them in DMEM/F12 (Solarbio, China) containing 10% fetal bovine serum (Gibco, Australia) and penicillin (100 U/ml)-streptomycin (100 μg/mL) (Solarbio, China) and then placed in an incubator at 37°C with 5% CO_2_. When 80% confluence was reached, the cells were detached with 0.25% EDTA-containing trypsin. The complete expansion medium was changed every other day. The NPCs of the first three passages were used in all experiments. During the experiments, the phenol red-free medium was used to avoid the weak estrogenic effects from phenol red.

The NPCs were cultured in 6-well- or 24-well plates and divided into the following groups, with three replicates in each group. The control group was treated with normal culture medium only. The IL-1β group was treated with IL-1β (75 ng/mL (Wang et al., 2021), SRP3083, Sigma-Aldrich) for 24 h. The E2 supplement group was pretreated with E2 (1 μM, HY-B0141, MCE) for 30 min, followed by IL-1β (75 ng/mL) treatment for 24 h. The PF group was firstly pretreated with PF4708671 (10 μM (Rosner et al., 2012; Pearce et al., 2010), HY-15773, MCE) for 30 min, and then treated with E2 (1 μM) for 30 min, followed by an incubation with IL-1β (75 ng/mL) for 24 h.

### 2.7 Western blot analysis

Protease inhibitors (1%) and phosphotransferase inhibitors (1%) (Solarbio, China) were added to RIPA lysate (Solarbio, China) before being used to lyse NPCs samples from 6-well plates. The total protein was extracted by centrifugation. The protein concentration was determined using a BCA protein assay kit (Pierce, 23,225). For gel electrophoresis, 10% or 12% SDS-PAGE gel was used. The electrophoresis ran for 70 min at a consistent voltage of 100 V. Next, membrane transfer was conducted using a PVDF membrane for 50 min at a consistent current of 400 mA. The membrane was blocked with a protein-free rapid-sealing solution for 30 min, followed by incubation with primary antibodies for overnight at 4°C.

The following primary antibodies were used: p70 S6K1 (1:4000, 14485-1-AP, Proteintech), Phospho-S6 (Ser240/244) (1:1000, D68F8, Cell Signaling Technology), MMP3 (1:1000, 17873-1-Ap, Proteintech), Aggrecan (1:1000, DF7561, Affinity), Collagen II (1:1000, AF0135, Affinity), cleaved caspase-3 (1:1000, 9661s, Cell Signaling Technology), GADPH(1:500, P04406, Abways), β-actin (1:10,000, 0,007,907, Proteintech).

Next, the membrane was rinsed by TBST, followed by an incubation with secondary antibodies for 1 h at room temperature. Finally, blots were detected using an ECL imager (ChemiDoc, BIO-RAD, USA). For semi-quantitative analysis, grayscale values were measured using ImageJ software (version 1.53k; National Institutes of Health, Bethesda, MD, USA).

### 2.8 Immunofluorescence staining

The NPCs were attached to a coverslip and cultured in a 24-well plate. After harvest, the NPCs were fixed with 4% PFA for 15 min at room temperature. After wash with PBS, the cells were blocked at room temperature for 1 h with donkey serum (Solarbio, China) which was diluted to 5% with PBST. Next, an overnight incubation was performed at 4°C with the following primary antibodies: p70 S6K1 (1:50, 14485-1-AP, Proteintech), Phospho-S6 (Ser240/244) (1:800, D68F8, Cell Signaling Technology), MMP3 (1:800, 17873-1-Ap, Proteintech), Aggrecan (1:200, DF7561, Affinity), Collagen II (1:200, AF0135, Affinity), cleaved caspase-3 (1:400, 9661s, Cell Signaling Technology). After being re-heated for 1h, the primary antibodies were removed, washed with PBS. The anti-rabbit IgG (Alexa Fluor 555-labeled donkey anti-rabbit IgG (H + L), 1:500, A0453, Beyotime) was added and incubated in the dark at room temperature for 2 h. Nuclear staining was performed with DAPI (Solarbio, China). Finally, all fluorescence images were acquired with identical exposure time and microscope settings to ensure comparability of fluorescence intensity across experimental groups. The images were collected using a confocal laser scanning microscope (Nikon, Tokyo, Japan). The average fluorescence intensity of NPCs in the images was analyzed using ImageJ software (version 1.53k; National Institutes of Health, Bethesda, MD, USA).

### 2.9 Statistical analysis

All data were analyzed and plotted using GraphPad Prism 8.2 (GraphPad Software, San Diego, CA, United States). The normality of data distribution was confirmed using the Shapiro-Wilk test (p > 0.05) followed by one-way analysis of variance (ANOVA) for statistical analysis among groups with the Bonferroni test. All data were presented as mean ± standard deviation. P < 0.05 was regarded as statistically significant.

## 3 Results

### 3.1 E2 alleviated IVDD progression and improved ECM homeostasis

**FIGURE 1:**
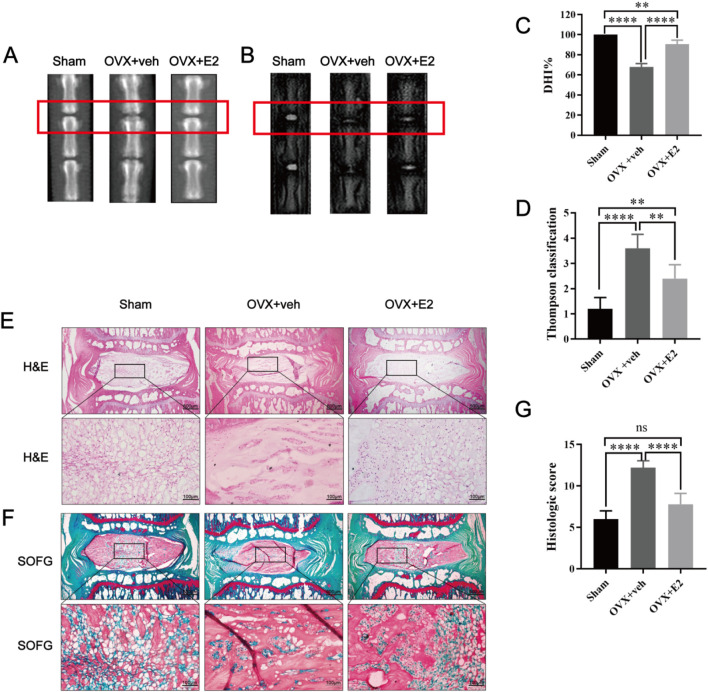
Rat IVDD model induced by needle puncture and OVX and histological staining. Rat IVD sections shown from left to right: sham ovariectomy (Sham), needle puncture plus ovariectomy with vehicle injection (OVX + Veh), and needle puncture plus ovariectomy with estradiol hormone replacement injection (OVX + E2). **(A, C)** Representative X-ray images of rat coccygeal vertebra and measurement result of DHI changes. DHI% was calculated as: DHI = 2 × (D1 + D2 + D3)/(V1 + V2 + V3+ V4+ V5 + V6), DHI% = post-punctured DHI/pre-punctured DHI × 100%, where D indicates disc height and V indicates vertebra length. **(B, D)** MRI images of rat coccygeal IVDs from different treatment groups and the scores of IVDD evaluated by the modified Thompson classification. **(E)** There are low-magnification H&E sections and higher magnification images of boxed areas. **(F)** Low magnification Safranin O-Fast green sections and higher magnification images of boxed areas. The green components indicate an acidophilic matrix stained by Fast green, and the red components indicate a basophilic matrix stained by Safranin O. **(G)** Histological scores for rat IVDs in the three groups above. Values are mean ± SD (n = 5). ns: not significant; **p < 0.01; ***p < 0.001; ****p < 0.0001. E2, 17β-estradiol; AF, annulus fibrosus; IVD, intervertebral disc; IVDD, intervertebral disc degeneration; OVX, ovariectomy; veh, vehicle injection; DHI, disc height index; MRI, magnetic resonance imaging; H&E, hematoxylin and eosin staining; SOFG, Safranin O-Fast Green staining; SD, standard deviation.

**FIGURE 2:**
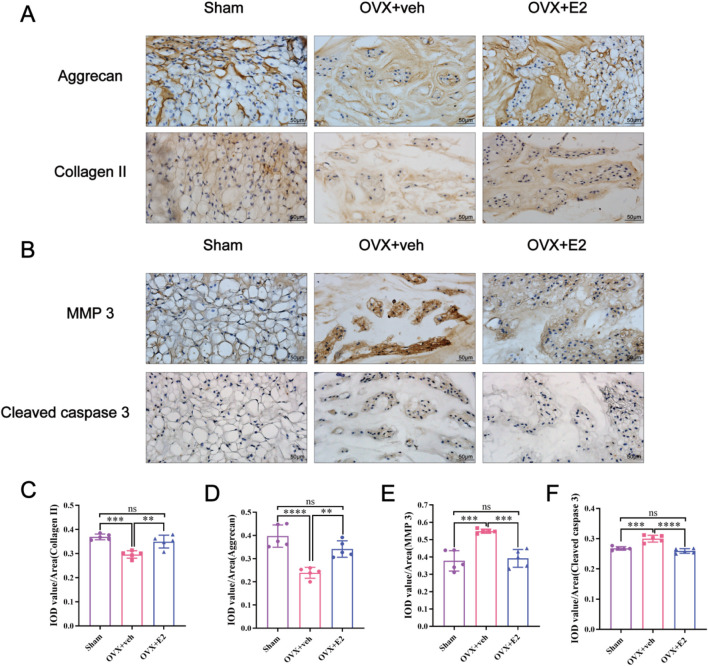
E2 inhibits MMPs and cleaved caspase 3 expression and reduces the ECM degradation. The rat IVD sections shown from left to right: sham ovariectomy (Sham), needle puncture plus ovariectomy with vehicle injection (OVX + Veh), and needle puncture plus ovariectomy with estradiol hormone replacement injection (OVX + E2) groups. **(A, C, D)** The expression of aggrecan and collagen II, which are the main components of ECM in NP tissues stained by immunohistochemical staining. **(B, E, F)** The expression of MMP 3 and cleaved caspase 3 in NP tissues stained by immunohistochemical staining. Values are mean ± SD (n = 5). ns: not significant; *p < 0.05; **p < 0.01; ***p < 0.001; ****p < 0.0001. IVD, intervertebral disc; OVX, ovariectomy; veh, vehicle injection; E2, 17β-estradiol; MMP, matrix metalloproteinase; ECM, extracellular matrix; NP, nucleus pulposus; SD, standard deviation.

As shown in Figure 1, X-ray images showed significant differences in disc height between the OVX + veh and OVX + E2 groups. DHI% in the OVX + veh group was significantly lower compared with the Sham and OVX + E2 groups (Figures 1A,C). MRI images showed that the signal intensity of the OVX + veh group was lower compared with the Sham and OVX + E2 groups. After E2 treatment, the IVD signal intensity of the OVX + E2 group was higher than the OVX + veh group. However, the signal was still weaker than the Sham group (Figures 1B,D). H&E and Safranin O-Fast Green staining showed that NPCs and ECM (aggrecan and collagen) were significantly reduced in the OVX + veh group. The NP and ECM structures of the OVX + E2 group were relatively complete and significantly better than those of the OVX + veh group (Figures 1E,F). In addition, histological scores were significantly higher in the OVX + veh group compared with the other two groups (Figure 1G). Immunohistochemical staining showed that the levels of aggrecan and collagen II in the Sham and OVX + E2 groups were significantly higher compared with the OVX + veh group (Figures 2A,C,D). The levels of MMP3 and Cleaved caspase 3 in the Sham and OVX + E2 groups were significantly lower compared with the OVX + veh group (Figures 2B,E,F).

### 3.2 E2 mitigated IVDD progression by activating the p70 S6K1 signaling pathway

**FIGURE 3:**
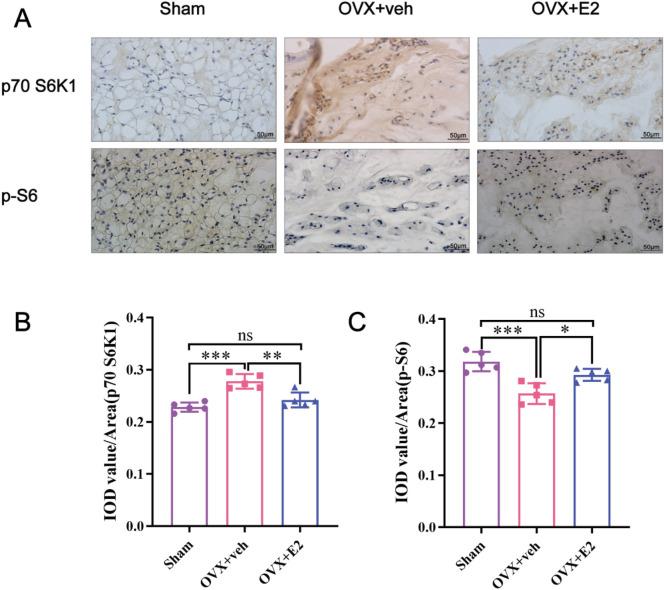
Immunohistochemistry showing that E2 mitigates IVDD by activating the p70 S6K1 signaling pathway downstream of mTOR *in vivo*. The rat IVD sections shown from left to right: sham ovariectomy (Sham), needle puncture plus ovariectomy with vehicle injection (OVX + Veh), and needle puncture plus ovariectomy with estradiol hormone replacement injection (OVX + E2) groups. **(A)** The levels of p70S6K1 and p-S6 in NP tissues of rat tails were detected using immunohistochemistry. **(B, C)** Data analysis of the immunohistochemistry. Values are mean ± SD (n = 5). ns: not significant; *p < 0.05; **p < 0.01; ***p < 0.001. IVD, intervertebral disc; IVDD, intervertebral disc degeneration; OVX, ovariectomy; veh, vehicle injection; E2, 17β-estradiol; NP, nucleus pulposus; p-S6, Phospho-S6 (the ribosomal protein S6); SD, standard deviation.

As shown in Figures 3A–C, the IHC staining demonstrated that compared with the Sham group, the level of p70 S6K1 was significantly increased in the OVX + veh group while the level of p-S6, the phosphorylated substrate downstream of p70 S6K1, was significantly decreased. After E2 treatment, the level of p70 S6K1 decreased in the OVX + E2 group while the level of p-S6 significantly increased (all P < 0.05). Collectively, these results indicated that E2 promoted the activation of the p70 S6K1 signaling pathway, downstream of mTOR during the progression of IVDD.

### 3.3 E2 activates p70 S6K1 signaling pathway of human NPCs *in vitro*

**FIGURE 4:**
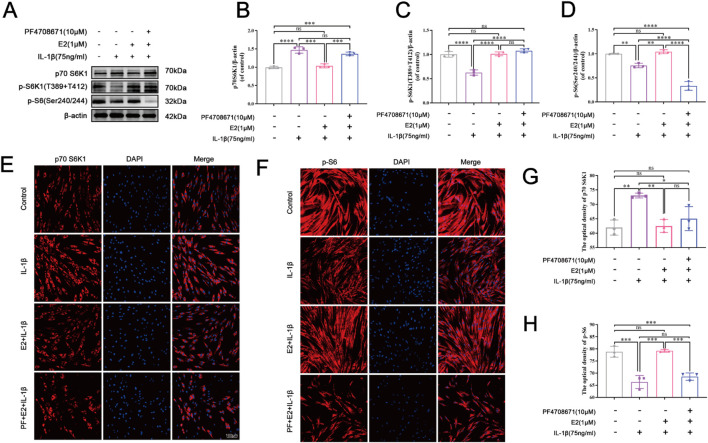
E2 activates the p70 S6K1 signaling pathway *in vitro*. **(A–D)** Western blotting was used to analyze the levels of p70 S6K1, p-S6K1 and p-S6 in human NPCs. **(E, G)** The level of p70 S6K1 in human NPCs by immunofluorescence staining. **(F, H)** The level of p-S6 in human NPCs by immunofluorescence staining. Values are mean ± SD (n = 3). ns: not significant; *p < 0.05; **p < 0.01; ***p < 0.001; ****p < 0.0001. E2, 17β-estradiol; NPCs, nucleus pulposus cells; IL-1β, interleukin-1β; PF, PF4708671 (an p70 S6K1 inhibitor); p-S6, Phospho-S6 (the ribosomal protein S6); SD, standard deviation.

To further explore the potential mechanism by which E2 mitigates the IVDD progression, the p70 S6K1 signaling pathway was investigated using IL-1β-induced human NPCs apoptosis model. As shown in Figure 4, Western blot analysis and cellular immunofluorescence staining were performed to determine the levels of p70 S6K1, p-S6K1, and p-S6. The p70 S6K1 is one of the downstream effect targets of mTOR, and the degradation of p70 S6K1 and phosphorylation of S6K1 and S6 proteins represent the activation of p70 S6K1 signaling pathway. Western blot analysis showed that compared with the control group, the level of p70 S6K1 in the IL-1β group was significantly increased, while the levels of p-S6K1 and p-S6 were significantly decreased (all P < 0.05). Compared with the IL-1β group, the level of p70 S6K1 decreased while the levels of p-S6K1 and p-S6 increased in the E2 group, suggesting that E2 can inhibit IL-1β-induced NPC apoptosis by activating the p70 S6K1 signaling pathway (Figures 4A–D).

The immunofluorescence staining indicated that the pretreatment of PF4708671 (10 μM) increased the level of p70 S6K1 compared to the E2 group, but there was no significance (Figures 4E,G). By contrast, the level of p-S6 was significantly decreased after the pretreatment of PF4708671 compared to the E2 group (P < 0.05) (Figures 4F,H). Taken together, these results suggest that E2 can protect NPCs by activating the p70 S6K1 signaling pathway.

### 3.4 E2 activation of p70 S6K1 signaling pathway maintains ECM homeostasis *in vitro*

**FIGURE 5:**
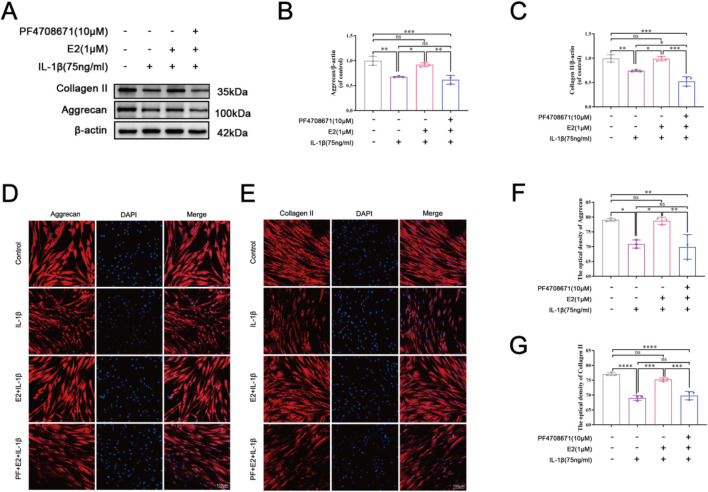
E2 activation of p70 S6K1 signaling pathway improves ECM anabolism *in vitro*. **(A–C)** Western blot analysis was used to analyze the expression of Collagen II and Aggrecan in human NPCs. **(D, F)** The expression of Aggrecan in human NPCs by immunofluorescence staining. **(E, G)** The expression of Collagen II in human NPCs by immunofluorescence staining. Values are mean ± SD (n = 3). ns: not significant; *p < 0.05; **p < 0.01; ***p < 0.001; ****p < 0.0001. E2, 17β-estradiol; NPCs, nucleus pulposus cells; IL-1β, interleukin-1β; PF, PF4708671(an p70 S6K1 inhibitor); ECM, extracellular matrix; SD, standard deviation.

As shown in Figure 5, Western blot and cell immunofluorescence staining showed that IL-1β significantly decreased the expression levels of aggrecan and collagen II which were upregulated by the pretreatment of E2. The addition of PF4708671, a specific p70 S6K1 pathway inhibitor, effectively downregulated the levels of aggrecan and collagen II.

**FIGURE 6:**
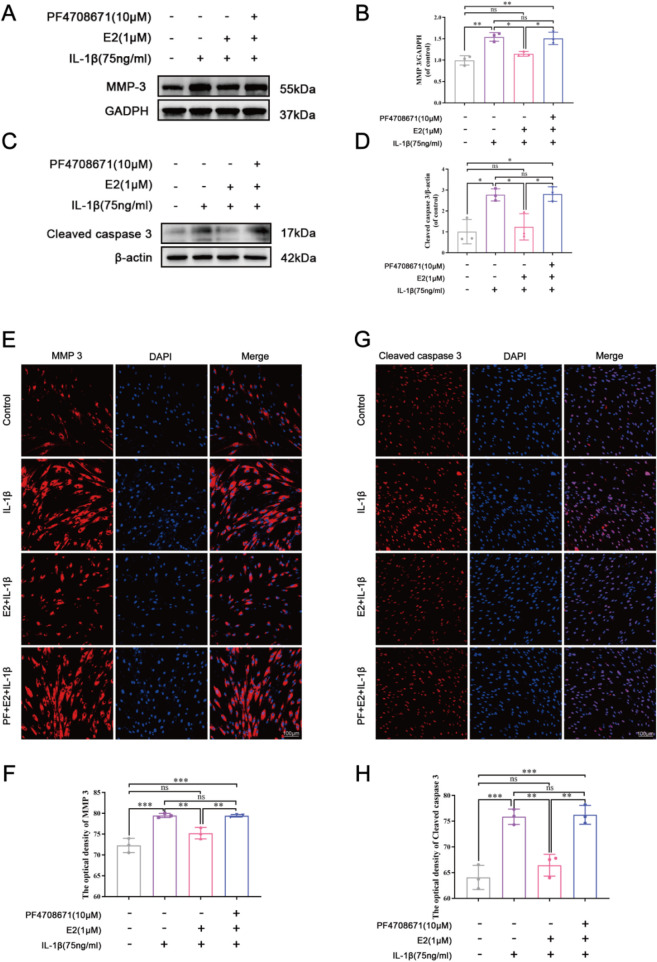
E2 activation of the p70 S6K1 signaling pathway inhibits MMP3 and cleaved caspase 3 *in vitro*. **(A–D)** Western blot analysis was used to analyze the expression of MMP3 and cleaved caspase 3. **(E, F)** The expression of MMP3 was determined by immunofluorescence staining. **(G, H)** The expression of cleaved caspase 3 was determined by immunofluorescence staining. Values are mean ± SD (n = 3). ns: not significant; *p < 0.05; **p < 0.01; ***p < 0.001. E2, 17β-estradiol; NPCs, nucleus pulposus cells; IL-1β, interleukin-1β; PF, PF4708671(an p70 S6K1 inhibitor); MMP3, matrix metalloproteinase 3; SD, standard deviation.

As shown in Figure 6, IL-1β significantly increased the expression levels of MMP3 and cleaved caspase 3, while E2 effectively reversed their levels. The effects of E2 can be abolished by the addition of PF4708671, a specific p70 S6K1 pathway inhibitor, which significantly increased the levels of MMP3 and cleaved caspase 3. These results together indicate that E2 maintains ECM homeostasis and inhibits NPC apoptosis by activating p70 S6K1 downstream of mTOR.

## 4 Discussion

In recent years, studies have found that IVDD is one of the leading potential causes of low back pain (Wang et al., 2021; Wang et al., 2023; Chen et al., 2017), which is a severe health problem that will reduce the quality of life. To date, the pathology and mechanism of IVDD have yet to be fully understood. Studies have found that postmenopausal women have a relatively high incidence of low back pain, which may be related to the decreased estrogen level in postmenopausal women (Wang, 2015; Wang, 2017). Some studies have also confirmed that E2 can inhibit the apoptosis of NPCs, thereby alleviating IVDD (Liu et al., 2018; Wang et al., 2021; Yang et al., 2015).

The mechanical/mammalian target of rapamycin (mTOR) is an evolutionarily conserved serine/threonine kinase and an important member of the phosphocreatine 3-kinase-associated kinase (PIKK) family (Mita et al., 2003). mTOR signal transduction-driven translation regulation is mainly dependent on eukaryotic translation initiation factor 4E (eIF4E) binding protein (4E-BP), ribosomal protein S6 kinase (S6K), and its downstream participants, which play an essential role in the rapid cellular response to environmental changes (Yang et al., 2022). It has been found that activated protein kinase S6K, one of mTOR’s downstream effectors, transfers upstream signals to various effectors to regulate a variety of cellular processes (Jastrzebski et al., 2009; Magnuson et al., 2012), such as inhibiting glycogen synthase kinase-3 (GSK3), Bcl-2/BCLXL-antagonists, causes cell death and activates the cAMP response element regulator τ (CREMτ) and the estrogen receptor (ERα) to promote transcription (Yang et al., 2022).

Meanwhile, relevant studies have shown that the PI3K/AKT/mTOR signaling pathway is involved in the progression of IVDD (Kakiuchi et al., 2019; Bai et al., 2021; Ito et al., 2017). However, the exact role of the p70 S6K1 signaling pathway, one of the downstream effect targets of mTOR, is unclear. Bai et al. (2021) reported that the combination of resveratrol and E2 effectively inhibited the apoptosis of NPCs induced by IL-1β *in vitro*, mainly through the PI3K/AKT/mTOR/caspase-3 and PI3K/AKT/GSK-3β pathways. This study suggests that E2 can inhibit NPCs apoptosis by activating the PI3K/AKT/mTOR pathway. However, there is no evidence of the expression of downstream mTOR pathways in human and rat NP tissues. By using immunohistochemical staining, our study found that the expression of p70S6K1 and its phosphorylated products in rat IVD changed with the degree of IVDD. Our *in vitro* study showed that the positive expression of p-S6 and p-S6K1 in the OVX + E2 group was higher than that in the OVX + veh group, while the positive expression of p70 S6K1 was lower, indicating that E2 had an activation effect on p70 S6K1 signaling pathway. Therefore, these results preliminarily demonstrate a close relationship between estrogen, p70 S6K1 signaling pathway downstream of mTOR, and IVDD.

It is well known that there are many causes of IVDD, including biomechanical factors, spinal instability, NPC apoptosis, and metabolic disorders of the ECM (Parreira et al., 2018). The ECM (including collagen, aggrecan, and other matrix proteins) maintains the trophic balance inside and outside of the IVD, and its metabolites may induce inflammation when the balance is disrupted (Wang et al., 2019; Wang et al., 2021; Chen et al., 2017). MMPs (MMP3 and MMP13, etc.) are the major collagen-degrading enzymes in the ECM and inhibit collagen II and aggrecan synthesis, further exacerbating the process of IVDD (Wang et al., 2019; Wang et al., 2023). Meanwhile, relevant studies have reported that apoptosis and inflammation of NPCs are key contributing factors to IVDD, which can accelerate the progression of IVDD by inducing the high expression of MMPs to degrade ECM (Wang et al., 2019; Ding et al., 2013; Risbud and Shapiro, 2014; Chen et al., 2017). Apoptosis is initiated and executed by the caspase family of cysteine proteases located in the cytoplasm. Downregulation of caspase-3 can prevent apoptosis and degenerative changes of the IVD (Wang et al., 2021; Li et al., 2019; Dai et al., 2021). Based on a previous study, we established rat IVDD model using bilateral ovariectomy combined with needle puncture to AF (Wang et al., 2021). Using histological and imaging analysis, we found that the estrogen therapy could reduce NPCs apoptosis and promote ECM balance by down-regulating the levels of MMP3 and cleaved caspase 3, thus effectively attenuating IVDD.

In the *in vitro* experiments, we investigated the effect of E2 on the p70 S6K1 signaling pathway using human NPCs. Compared with the IL-1β group, it was found that E2 can significantly decrease the level of p70 S6K1 while increase the expression of its phosphorylated substrates p-S6 and p-S6K1, indicating that this pathway was activated by E2. When using a p70 S6K1 signaling pathway inhibitor (PF4708671), we found that p70 S6K1 expression increased, and its phosphorylated substrate p-S6 expression decreased compared to the E2 group. However, p-S6K1 is highly expressed because PF-4708671 can block the activity of p70 S6K1 kinase and induce its phosphorylation at T389, which mainly inhibits its downstream phosphorylation substrate p-S6 (Rosner et al., 2012). Meanwhile, the effects of the p70 S6K1 pathway on the expression of MMP3, cleaved caspase 3, Collagen II, and aggrecan were further examined through cell experiments. Our results showed that inhibition of the p70 S6K1 pathway in the IL-1β and PF groups increased the expression of MMP3 and cleaved caspase 3, resulting in the degradation of ECM-the reduction of collagen II and aggrecan. On the contrary, the results of the control group and E2 group were similar to those of previous studies (Liu et al., 2018; Wang et al., 2021; Yang et al., 2015). These results further suggest that E2 can improve IVDD by activating the p70 S6K1 signaling pathway, down-regulating the expression of MMP3 and cleaved caspase 3, and restoring ECM balance.

There are some limitations in this study. First, we verified the protective effect of E2 on IL-1β-induced apoptosis of NP cell lines. However, the impact of E2 on human NPCs isolated from clinical patients has not been validated. Secondly, only ovariectomized female rats were selected in our study as the study objects. In addition, we selected 3-month-old rats, not old rats, which may impact the experimental results. Finally, the downstream targets of the mTOR pathway mainly include S6K and 4E-BP, which are involved in translation control. S6K consists of S6K1 and S6K2, but mainly, S6K1 is activated to transmit signals to various effector factors to regulate multiple cellular processes. There are three homologs of 4E-BP in mammals: 4E-BP1, 4E-BP2, and 4E-BP3. The regulatory mechanism mediated by 4E-BP is relatively more complex compared to S6K. Thus, this study only confirmed the p70 S6K1 signaling pathway downstream of mTOR, and the mechanism of the 4E-BP1 signaling pathway downstream of mTOR remains to be further studied.

## 5 Conclusion

In this study, we demonstrate that 17β-estradiol inhibits IL-1β-induced NPCs apoptosis and ECM degradation by activating the p70 S6K1 signaling pathway downstream of mTOR, thereby exerting its protective effect on intervertebral disc.

## Data Availability

The raw data supporting the conclusions of this article will be made available by the authors, without undue reservation.
